# Syntrophy via Interspecies H_2_ Transfer between *Christensenella* and *Methanobrevibacter* Underlies Their Global Cooccurrence in the Human Gut

**DOI:** 10.1128/mBio.03235-19

**Published:** 2020-02-04

**Authors:** Albane Ruaud, Sofia Esquivel-Elizondo, Jacobo de la Cuesta-Zuluaga, Jillian L. Waters, Largus T. Angenent, Nicholas D. Youngblut, Ruth E. Ley

**Affiliations:** a Department of Microbiome Science, Max Planck Institute for Developmental Biology, Tübingen, Germany; b Center for Applied Geosciences, Eberhard-Karls-Universität Tübingen, Tübingen, Germany; Rutgers University

**Keywords:** human gut, methanogens, microbiome, syntrophy

## Abstract

Across human populations, 16S rRNA gene-based surveys of gut microbiomes have revealed that the bacterial family *Christensenellaceae* and the archaeal family *Methanobacteriaceae* cooccur and are enriched in individuals with a lean, compared to an obese, body mass index (BMI). Whether these association patterns reflect interactions between metabolic partners, as well as whether these associations play a role in the lean host phenotype with which they associate, remains to be ascertained. Here, we validated previously reported cooccurrence patterns of the two families and their association with a lean BMI with a meta-analysis of 1,821 metagenomes derived from 10 independent studies. Furthermore, we report positive associations at the genus and species levels between *Christensenella* spp. and Methanobrevibacter smithii, the most abundant methanogen of the human gut. By coculturing three *Christensenella* spp. with M. smithii, we show that *Christensenella* spp. efficiently support the metabolism of *M. smithii* via H_2_ production far better than Bacteroides thetaiotaomicron does. Christensenella minuta forms flocs colonized by *M. smithii* even when H_2_ is in excess. In culture with C. minuta, H_2_ consumption by *M. smithii* shifts the metabolic output of *C. minuta*’s fermentation toward acetate rather than butyrate. Together, these results indicate that the widespread cooccurrence of these microorganisms is underpinned by both physical and metabolic interactions. Their combined metabolic activity may provide insights into their association with a lean host BMI.

## Abstract (precis)

The human gut microbiome is made of trillions of microbial cells, most of which are *Bacteria*, with a subset of *Archaea*. The bacterial family *Christensenellaceae* and the archaeal family *Methanobacteriaceae* are widespread in human guts. They correlate with each other and with a lean body type. Whether species of these two families interact and how they affect the body type are unanswered questions. Here, we show that species within these families correlate with each other across people. We also demonstrate that particular species of these two families grow together in dense flocs, wherein the bacteria provide hydrogen gas to the archaea, which then make methane. When the archaea are present, the ratio of bacterial products (which are nutrients for humans) is changed. These observations indicate that when these species grow together, their products have the potential to affect the physiology of their human host.

## INTRODUCTION

Obesity was the first human disease phenotype to be associated with an altered microbial ecology of the gut (1, 2). The link between the relative abundance in the gut of the bacterial family *Christensenellaceae* and a low host body mass index (BMI) now stands as one of the most robust associations described between the human gut microbiome and host BMI (3–15). Compared to other families of bacteria that comprise the human gut microbiome, the family *Christensenellaceae* was described relatively recently, when the type strain, Christensenella minuta, was reported in 2012 (16). Prior to the description of C. minuta, 16S rRNA sequences from this genus escaped notice in the gut microbiome, though these sequences accumulated steadily in small-subunit (SSU) rRNA gene databases. A positive association between a lean host BMI and the relative abundance in the gut of *Christensenellaceae* 16S rRNA genes was first reported in 2014 (4). The association was shown to have existed in earlier data sets (4) but was likely undetected, as this family had not yet been named. Goodrich et al. showed a causal link between the *Christensenellaceae* and host BMI in gnotobiotic mice: the addition of *C. minuta* to the gut microbiome of an obese human donor prior to transplantation reduced adiposity gains in the recipient mice compared to those of controls receiving the unsupplemented microbiome (4). The mechanism underlying this host response remains to be elucidated. One step toward this goal is a better understanding of how the members of the *Christensenellaceae* interact ecologically with other members of the gut microbiome.

Across human populations, the gut microbiota often forms patterns of cooccurrence (e.g., when these consortia exist in a subset of human subjects, they are termed enterotypes [17]). Such cooccurrences of taxa across subjects reflect shared environmental preferences, but to determine if they represent metabolic or physical interactions requires further study. The family *Christensenellaceae* consistently forms the hub of cooccurrence networks with other taxa (6, 8, 9, 18, 19). Notably, gut methanogens (specifically, of the archaeal family *Methanobacteriaceae*) are often reported as part of the *Christensenellaceae* cooccurrence consortium (4, 20–22). The most widespread and abundant of the gut methanogens, Methanobrevibacter smithii, produces CH_4_ from H_2_ and CO_2_, the products of bacterial fermentation of dietary fibers. Such cross-feeding likely explains why the relative abundances of M. smithii and fermenting bacteria are often positively correlated (21, 23, 24). Several studies have shown that in the laboratory, *M. smithii* can grow from the H_2_ provided by Bacteroides thetaiotaomicron, a common gut commensal bacterium (25–27). Given that the cultured representatives of the *Christensenellaceae* ferment simple sugars (16, 28) and that their genomes contain hydrogenases (29), we predicted that members of the *Christensenellaceae* produce H_2_ used by *M. smithii* as a substrate in methanogenesis.

Here, we explored the association between the *Christensenellaceae* and the *Methanobacteriaceae* in two ways. First, we analyzed metagenomes for statistical associations between the two families and their subtaxa. Compared to 16S rRNA gene surveys, metagenomes often can better resolve the taxonomic assignments of sequence reads below the genus level (30). Metagenome-based studies have so far been blind to the *Christensenellaceae*, however, because their genomes have been lacking from reference databases. Here, we customized a reference database to include *Christensenellaceae* genomes, which we used in a meta-analysis of >1,800 metagenomes from 10 studies. Second, to assess for metabolic interactions between members of the *Christensenellaceae* and *Methanobacteriaceae*, we measured methane production by *M. smithii* when grown in coculture with *Christensenella* spp. Our results show that (i) the positive association between the *Christensenellaceae* and the *Methanobacteriaceae* is robust to the genus/species level across multiple studies, (ii) these taxa associate with a lean host BMI, (iii) *Christensenella* spp. support the growth of *M. smithii* by interspecies H_2_ transfer far better than B. thetaiotaomicron does, and (iv) *M. smithii* directs the metabolic output of *C. minuta* toward less butyrate and more acetate and H_2_, which is consistent with reduced energy availability to the host and consistent with the association with a low BMI.

## RESULTS

### *Christensenella* relative abundance is significantly correlated with leanness across populations.

**FIG 1:**
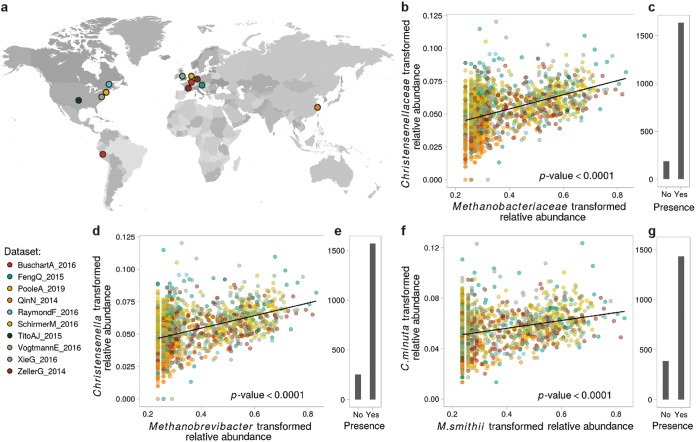
Abundances of the *Methanobacteriaceae* and *Christensenellaceae* families across populations. (a) Countries where the human gut metagenomes used in our meta-analysis (*n* = 1,821 samples) were recruited by 10 independent studies (summarized in Table S3); (b) association between the transformed relative abundances of *Christensenellaceae* and *Methanobacteriaceae* in samples where the a member of the *Methanobacteriaceae* was detected; (c) numbers of samples in which the *Methanobacteriaceae* were detected; (d and e and f and g) same as panels b and c at the genus and species levels, respectively. The correlation between the transformed relative abundances of both taxa at each taxonomic level was evaluated using linear mixed models to correct for covariates (ANOVA, *P* values < 0.0001).

Both the *Christensenellaceae* family and the genus *Christensenella* had very high prevalences, as they were present in more than 99% of the 1,821 samples; both the family and the genus have a mean abundance of 0.07% ± 0.05% (Fig. 1b and d and see Fig. S1 in the supplemental material). To correct for the influence of environmental factors on the relative abundances of members of the *Christensenellaceae* family and of the *Christensenella* genus, we first constructed null models in which we selected covariates (see Appendix 1 in Text S1) that explained a significant proportion of the variance of the transformed relative abundance of the family *Christensenellaceae* and in the same manner as that of the *Christensenella* genus. BMI and age were significantly correlated with the transformed relative abundances of members of the *Christensenellaceae* family and of the *Christensenella* genus (Cf-tra and Cg-tra, respectively, where the suffix “-tra” indicates transformed abundances) and were retained in the null models (Cf-null and Cg-null).

10.1128/mBio.03235-19.1 FIG S1 Abundances of the *Methanobacteriaceae* and *Christensenellaceae* families across studies. (a to j) Transformed relative abundances of *Christensenellaceae* (Cf-tra) and *Methanobacteriaceae* (Mf-tra) across 1,821 samples from 10 countries and generated from 10 independent studies. The data generated for this study are grouped with the first time series published in the work of Poole et al. (53). The gap between 0 and ∼0.2 is due to the detection limit of the sequencing method; the minimal relative abundance is 10^−3^%. Hence, 0 indicates that the microorganism was not detected, which introduces a gap after transformation of the data. Download FIG S1, PDF file, 2.6 MB. Copyright © 2020 Ruaud et al. 2020 Ruaud et al. This content is distributed under the terms of the Creative Commons Attribution 4.0 International license.

10.1128/mBio.03235-19.8 TEXT S1 Supplemental appendices. Download Text S1, DOCX file, 0.01 MB. Copyright © 2020 Ruaud et al. 2020 Ruaud et al. This content is distributed under the terms of the Creative Commons Attribution 4.0 International license.

10.1128/mBio.03235-19.7 TABLE S3 Data sets used for the statistical analysis. The BMI and age values for each data set are reported as average values, with minimum and maximum values in parentheses. Download Table S3, PDF file, 0.1 MB. Copyright © 2020 Ruaud et al. 2020 Ruaud et al. This content is distributed under the terms of the Creative Commons Attribution 4.0 International license.

BMI was negatively correlated with both Cf-tra (type II analysis of variance [ANOVA], *P* value = 0.0002 and *F* value [339] = 14.46) and Cg-tra (type II ANOVA, *P* value = 0.0002 and *F* value [339] = 14.29), indicating that leaner individuals harbor higher relative abundances of *Christensenellaceae* and *Christensenella*. Age was negatively correlated with Cf-tra (type II ANOVA, *P* value = 0.01 and *F* value [1,468] = 6.56) and with Cg-tra (type II ANOVA, *P* value = 0.01 and *F* value [1,468] = 6.53), indicating that younger subjects carry greater relative abundances of *Christensenellaceae* and *Christensenella*. However, the interaction term between BMI and age was not significantly correlated with the transformed relative abundances (type II ANOVA, *P* values > 0.1), indicating that their effects are additive. These results show that regardless of their BMIs, younger subjects have higher levels of *Christensenellaceae* and *Christensenella* and that the lower a subject’s BMI, the more of these microbes they harbor, regardless of their age.

### *Methanobrevibacter* relative abundance is significantly correlated with leanness across populations.

The *Methanobacteriaceae* family and *Methanobrevibacter* genus also had high prevalences, with 92% and 89% of people harboring them, respectively, and with mean abundances of 0.48% ± 1.55% and 0.49% ± 1.54%, respectively. As described above, we evaluated the association between the *Methanobacteriaceae* family and of the *Methanobrevibacter* genus by using models for BMI and age (models Mf-null and Mg-null). The transformed relative abundances of *Methanobacteriaceae*, Mf-tra, and of *Methanobrevibacter*, Mg-tra, were also negatively correlated with BMI (type II ANOVA, respective *P* values = 0.01 and 0.02, *F* values [341, 341] = 6.66 and 5.11, respectively). In contrast to the *Christensenellaceae*, both *Methanobacteriaceae* and *Methanobrevibacter* were positively correlated with age (type II ANOVA, respective *P* values = 0.001 and 4.27 × 10^−4^ and *F* values [1,468, 1,468] = 10.35 and 12.47), indicating that older people carry a greater proportion of methanogens. Moreover, *M. smithii*, the most abundant and prevalent methanogen species within the human gut, was also positively correlated with age and negatively with BMI regardless of age; i.e., the interaction term between age and BMI was not significantly correlated (see Appendix 2 in Text S1 for additional statistics).

### The relative abundances of the *Christensenella* and *Methanobrevibacter* genera are significantly correlated across populations.

Next, we looked into how the *Christensenellaceae* and the *Methanobacteriaceae* correlated with each other across subjects while controlling for BMI and age. We constructed a model where Mf-tra was included in addition to BMI and age (model Cf-Mf). This allowed us to test whether adding Mf-tra to the model improved its fit and, if so, how much of the variance of Cf-tra not explained by age and BMI could be explained by Mf-tra. We also evaluated the interaction terms between Mf-tra and BMI and between Mf-tra and age to assess whether the correlation between Cf-tra and Mf-tra was dependent on age and BMI. The interaction term for BMI and Mf-tra was not significant and was removed from the model; the interaction term for age and Mf-tra was significant and was retained (type I ANOVA, *F* value [339] = 8.30 and *P* value = 0.0042). We compared the log likelihoods of the null and full models (Cf-null and Cf-Mf) to confirm that the relative abundances of the *Methanobacteriaceae* and *Christensenellaceae* families were significantly correlated (χ^2^ test, *P* value = 1.78 × 10^−59^). Furthermore, the model Cf-Mf showed that Mf-tra was significantly positively correlated with Cf-tra (Fig. 1b) (type I ANOVA, *F* value [339] = 287.03, *P* value < 0.0001) and that the interaction term between Mf-tra and age was positively correlated with Cf-tra as well. These results indicate that the relative abundances of the *Christensenellaceae* and *Methanobacteriaceae* families are positively correlated across multiple populations/studies. In addition, although both families are enriched in low-BMI people, they are correlated regardless of a subject’s BMI. Moreover, their association is stronger in older people, suggesting that although elders are less likely to carry as much *Christensenellaceae* as youths, the more *Methanobacteriaceae* they have, the more *Christensenellaceae* they have.

We performed a similar analysis using the abundances of the two most prominent genera belonging to these families (*Christensenella* and *Methanobrevibacter*, models Cg-null and Cg-Mg) and obtained equivalent results. First, the interaction term between Mg-tra and age was positively correlated with Cg-tra (Fig. 1c) (type I ANOVA, *F* value [339] = 10.19, *P* value = 0.0015). Then, by comparing model Cg-null with our full model Cg-Mg, we showed that the relative abundances of the two genera were also correlated (χ^2^ test, *P* value = 1.50 × 10^−57^). Our full model Cg-Mg showed that Mg-tra was significant for predicting Cg-tra while controlling for BMI and age (type I ANOVA, *F* value [339] = 274.35, *P* value < 0.0001), with the interaction term between Mg-tra and age also positively correlating with Cg-tra. These results indicate that the correlations between the relative abundances of the two families, explained above, hold true at the level of two representative genera. The association between *Methanobrevibacter* and *Christensenella* is stronger in older people regardless of BMI.

A similar analysis at the species level indicated that *C. minuta* and *M. smithii* were the most abundant species of each of their genera, and similarly to the family and genus ranks, their relative abundances across samples were significantly correlated (Fig. 1d and Appendix 2 in Text S1). The less abundant *Christensenella* gut species C. massiliensis and C. timonensis also correlated with Methanobrevibacter smithii across the 1,821 metagenomes (see Appendix 2 in Text S1). *C. minuta* and *C. timonensis*’s transformed relative abundances were significantly negatively correlated with both BMI and age, while *C. massiliensis*’s transformed relative abundance was significantly correlated with BMI but not with age. Leaner people are thus enriched in members of the *Christensenellaceae* family, and *C. minuta* and *C. timonensis* are more abundant in young people than in older people.

### *C. minuta* forms flocs alone and in coculture with *M. smithii*.

**FIG 2:**
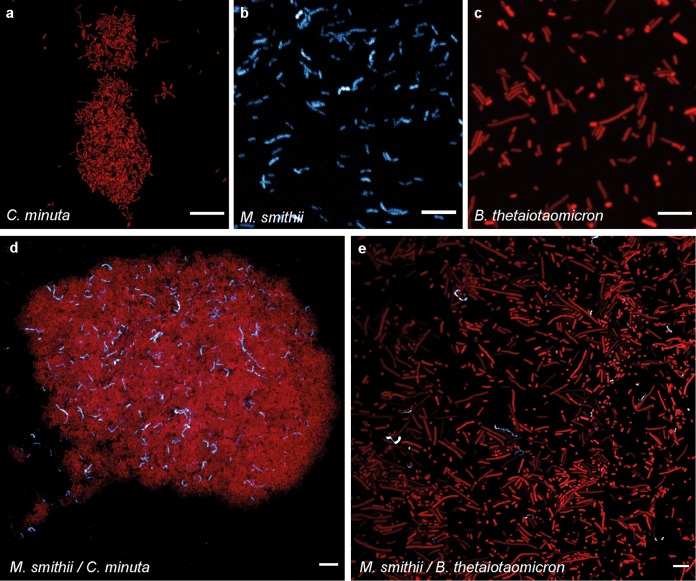
Confocal micrographs of the cultures after 3 days of growth. (a) *C. minuta* alone; (b) *M. smithii* alone; (c) B. thetaiotaomicron alone; (d) *M. smithii* and *C. minuta* together; (e) *M. smithii* and B. thetaiotaomicron together. SYBR green I fluorescence (DNA staining) is shown in red, and *M. smithii*’s coenzyme F_420_ autofluorescence is shown in blue. Scale bars represent 10 μm. Based on gas production, at 3 days of growth, B. thetaiotaomicron was already at stationary phase (explaining the elongated cells) (see Fig. S2 for confocal micrographs of B. thetaiotaomicron and *M. smithii* at 2 days of growth), *C. minuta* was at the end of the exponential phase, and *M. smithii* was still in exponential phase.

**FIG 3:**
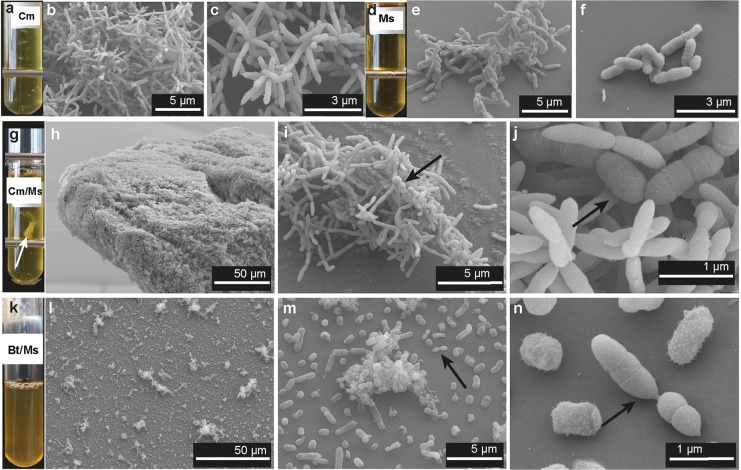
Scanning electron micrographs of the cultures at 3 to 7 days of growth. (a, d, g, and k) Representative Balch tubes of cultures of *C. minuta* (Cm), *M. smithii* (Ms), *C. minuta* and *M. smithii* (Cm/Ms), and B. thetaiotaomicron and *M. smithii* (Bt/Ms) after 7 days of growth; (b and c) scanning electron micrographs (SEMs) of monocultures of *C. minuta* at 5 days of growth; (e and f) SEMs of monocultures of *M. smithii* at 5 days of growth; (h to j) SEMs of cocultures of *C. minuta* and *M. smithii* at 7, 5, and 2 days of growth, respectively; (l to n) SEMs of cocultures of B. thetaiotaomicron and *M. smithii* at 7 days of growth. The floc formed by *C. minuta* and *M. smithii* is indicated with a white arrow in panel g; other arrows indicate *M. smithii* cells. Metal bars in panels a, d, and g are from the tube rack.

To assess the physical and metabolic interactions of two representative species, we used *C. minuta* DSM-22607, previously shown to reduce adiposity in germfree mouse fecal transplant experiments (4), and *M*. *smithii* DSM-861, which is the most abundant and prevalent methanogen in the human gut (31). Confocal and scanning electron imaging of 2- to 7-day-old cultures revealed that *C. minuta* organisms flocculate in mono- and cocultures (Fig. 2a and b and Fig. 3a to c and g to j). *M. smithii* is present within the *C. minuta* flocs (Fig. 2d and Fig. 3g to j) but does not aggregate in monoculture before 7 to 10 days of culture (data not shown). In contrast, B. thetaiotaomicron, used here as a positive control based on previous reports that it supports the growth of *M. smithii* via H_2_ production (25, 26), did not flocculate when grown alone (Fig. 2c) and, when cocultured with *M*. *smithii*, displayed very limited aggregation (Fig. 2e, Fig. 3k to n, and Fig. S2).

**FIG 4:**
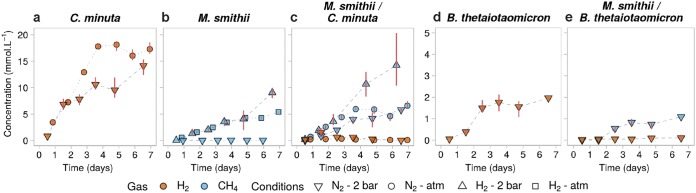
Gas concentrations over time in mono- and cocultures of *C. minuta*, B. thetaiotaomicron, and *M. smithii* grown under different conditions. (a to e) H_2_ (orange) and CH_4_ (blue) concentrations in the headspace over time in cultures from batches 1 to 3 (Table S1). Points represent the averages of results from 3 biological replicates for each condition, and red bars join the minimal and maximal values. In conditions where H_2_ was provided in excess (H_2_, 2 × 10^5^ Pa, and atmospheric (atm) H_2_, with the headspace initially composed of 80:20, vol/vol, H_2_-CO_2_), its concentrations are not shown for scale reasons. Initial concentrations of H_2_ under conditions where it was not provided in the headspace were undetectable (N_2_, 2 × 10^5^ Pa, and atmospheric N_2_, with the headspace initially composed of 80:20, vol/vol, N_2_-CO_2_) and stayed null in the monocultures of *M. smithii* (not shown). CH_4_ concentrations in the bacterial monocultures were undetectable and are not shown as well. (a to c) Same *y* scale as in panels d and e.

10.1128/mBio.03235-19.2 FIG S2 Confocal imaging of cocultures of B. thetaiotaomicron and *M. smithii* at different time points. (a and b) Cells at day 2, when B. thetaiotaomicron enters stationary phase (Fig. 4d); (c and d) cells at day 7, the end of the experiment, when maximal CH_4_ concentrations were observed both in monocultures of *M. smithii* and in cocultures with B. thetaiotaomicron (Fig. 4b and e). In exponential phase, B. thetaiotaomicron cells are rod shaped (a), while during stationary phase, they suffer stress, leading to elongated cells (c). The bright fields (a and c) and *M. smithii*’s coenzyme F_420_ (b and d) channels are displayed. Scale bars represent 10 μm. Download FIG S2, JPG file, 0.1 MB. Copyright © 2020 Ruaud et al. 2020 Ruaud et al. This content is distributed under the terms of the Creative Commons Attribution 4.0 International license.

### H_2_ and CH_4_ production.

**FIG 5:**
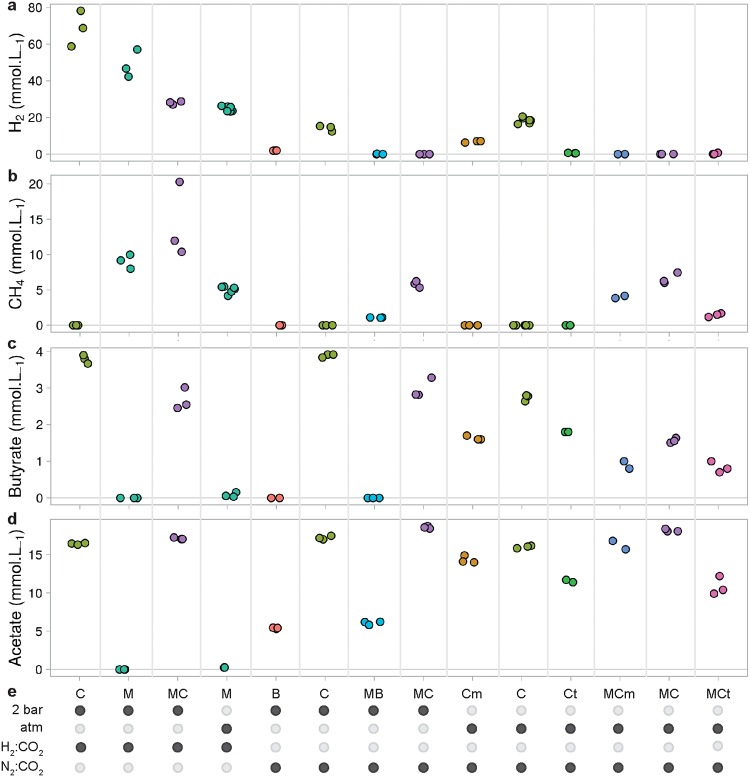
Summary of gases and SCFAs produced in mono- and cocultures of *C. minuta*, *C. timonensis*, *C. massiliensis*, B. thetaiotaomicron, and *M. smithii* after 6 days of growth. (a to d) H_2_, CH_4_, butyrate, and acetate production after 6 days of growth in all mono- and cocultures presented in this study (batches 1 to 4) (Table S1). Points represent the concentration of each biological replicate. (e) Table summarizing the conditions for each culture. The conditions include the gas mixture (H_2_-CO_2_ or N_2_-CO_2_ at 80:20, vol/vol), the initial pressure (2 × 10^5^ Pa or atmospheric), and the microorganisms inoculated. C, *C. minuta*; Ct, *C. timonensis*; Cm, *C. massiliensis*; B, B. thetaiotaomicron; M, *M. smithii*; MC, *M. smithii* and *C. minuta*; MB, *M. smithii* and B. thetaiotaomicron; MCm, *M. smithii* and *C. massiliensis*; MCt, *M. smithii* and *C. timonensis*. Samples inoculated with the same microorganisms are the same color.

After 6 days in monoculture, *C. minuta* had produced 7 times more H_2_ than B. thetaiotaomicron (14.2 ± 1.6 mmol · liter^−1^ versus 2.0 ± 0.0 mmol · liter^−1^) (Fig. 4a and d and Fig. 5a; Wilcoxon rank sum test, *P* value = 0.1). As expected, *M. smithii* did not grow in monoculture when H_2_ was not supplied (80:20, vol/vol, N_2_-CO_2_ headspace) (Fig. 4b). After 6 days, *M. smithii* had produced 9.0 ± 1.0 mmol · liter^−1^ of CH_4_ when H_2_ was provided in excess (i.e., 80:20, vol/vol, H_2_-CO_2_ atmosphere at 2 × 10^5^ Pa) (Fig. 4b and Fig. 5b).

10.1128/mBio.03235-19.5 TABLE S1 Total pressure, headspace composition, and culture inocula for each batch of experiments described in the main text. Download Table S1, PDF file, 0.02 MB. Copyright © 2020 Ruaud et al. 2020 Ruaud et al. This content is distributed under the terms of the Creative Commons Attribution 4.0 International license.

In accordance with the higher levels of H_2_ produced by *C. minuta* than by B. thetaiotaomicron, day 6 CH_4_ concentrations were higher for *M. smithii* cocultured with *C. minuta* than with B. thetaiotaomicron (respectively, 5.8 ± 0.5 mmol · liter^−1^ and 1.1 ± 0.0 mmol · liter^−1^; Wilcoxon rank sum test, *P* value = 0.1) (Fig. 4c and e and Fig. 5b). For both coculture conditions, H_2_ concentrations were very low (on average, across time points and replicates, H_2_ concentrations were 0.5 ± 0.6 mmol · liter^−1^ in cocultures with *C. minuta* and 0.1 ± 0.1 mmol · liter^−1^ in cocultures with B. thetaiotaomicron), indicating that almost all the H_2_ that had been produced was also consumed (Fig. 4c and e and Fig. 5a).

### Pressure effects on gas production and aggregation.

Gas-consuming microbes, including hydrogenotrophic methanogens, grow better in a pressurized environment (32–34) due to a higher gas solubility at higher pressure, as described by Henry’s law. We compared levels of CH_4_ production by *M. smithii* in monoculture and in coculture with *C. minuta* under 2 different pressures (i.e., 2  × 10^5^ Pa and atmospheric pressure). As with their flocculation at 2 × 10^5^ Pa (Fig. 2d), *C*. *minuta* and *M. smithii* aggregated at atmospheric pressure (Fig. S3a and b). Accordingly, *C. minuta* supported CH_4_ production by *M*. *smithii* to similar extents under both pressure conditions (ANOVA followed by Tukey’s *post hoc* test, adjusted *P* value = 1.0) (Fig. 4c and 5b), even though the putative H_2_ produced by *C. minuta* (estimated based on the monocultures) was much lower than the amount of H_2_ provided in the headspace for *M. smithii* (Fig. 5a).

10.1128/mBio.03235-19.3 FIG S3 *C. minuta* and *M. smithii* aggregate at atmospheric pressure and even when there is excess H_2_ in the medium. Confocal imaging of *C. minuta* and *M. smithii* at 3 days of growth. (a and b) Coculture grown at atmospheric pressure; (c and d) coculture grown under a pressurized H_2_-CO_2_ atmosphere. The bright fields (a and c) and *M. smithii*‘s coenzyme F_420_ (b and d) channels are displayed. Scale bars represent 10 μm. Download FIG S3, JPG file, 0.2 MB. Copyright © 2020 Ruaud et al. 2020 Ruaud et al. This content is distributed under the terms of the Creative Commons Attribution 4.0 International license.

We next sought to assess whether the mixed aggregation of *M. smithii* with *C. minuta* could be disrupted if H_2_ was pressurized in the medium, reducing *M. smithii*’s reliance on *C. minuta* as a H_2_ source. We observed that *M. smithii* aggregated with *C. minuta* (Fig. S3c and d) even though H_2_ was abundant. Total CH_4_ production was higher than in monocultures under the same headspace, reaching 14.2 ± 5.3 mmol · liter^−1^ in coculture versus 9.0 ± 1.0 mmol · liter^−1^ in monoculture after 6 days (ANOVA followed by Tukey’s *post hoc* test, adjusted *P* value = 0.1) (Fig. 4b and c). This indicates that interspecies H_2_ transfer occurs even when H_2_ is added to the headspace and leads to greater methanogenesis.

### The SCFA production of *C*. *minuta* is influenced by the presence of *M*. *smithii*.

Regardless of headspace composition and pressure conditions, the only short-chain fatty acids (SCFAs) detected as produced by *C. minuta* in monoculture were acetate and butyrate (among 10 short and medium-chain fatty acids analyzed) (see Appendix 1 in Text S1 and Fig. 5). To investigate if the consumption of H_2_ by *M. smithii* influenced the SCFA production profile of *C. minuta*, we compared acetate and butyrate concentrations between the cocultures and *C. minuta*’s monocultures under all conditions tested (i.e., cultures at 2 × 10^5^ Pa or atmospheric pressure with an 80:20, vol/vol, N_2_-CO_2_ or H_2_-CO_2_ headspace (Table S1).

**FIG 6:**
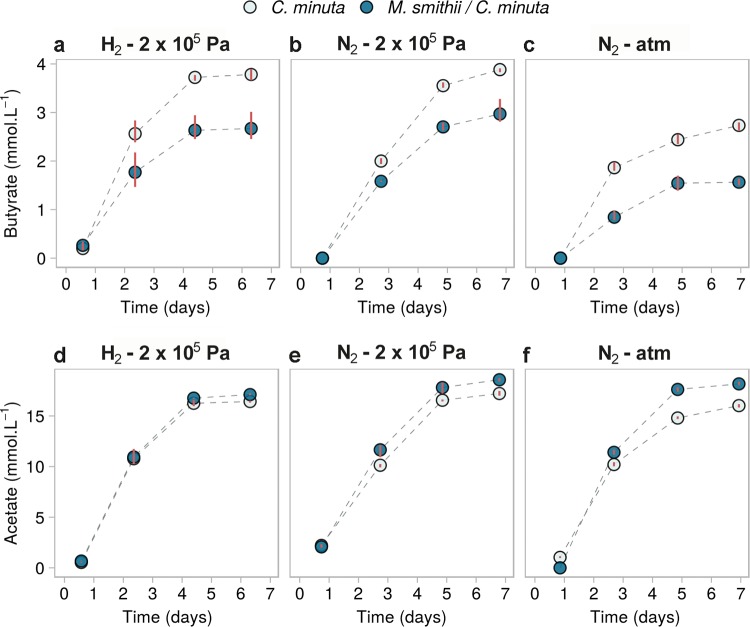
SCFA concentrations over time in mono- and cocultures of *C. minuta* and *M. smithii* grown under different conditions and in cultures from batches 1 to 3 (Table S1). (a to c) Butyrate concentrations; (d to f) acetate concentrations. Only these SCFAs were detected among the fatty acids tested (fatty acids from C_1_ to C_8_, iso-valerate, and iso-butyrate). Points represent the averages of results from 3 biological replicates for each condition, and red bars join the minimal and maximal values. Monocultures of *M. smithii* are not shown, as they did not differ from the blanks (negative controls).

We consistently observed lower butyrate concentrations in all cocultures than in monocultures (Fig. 6a to c and 5c) (ANOVA, *F* value [1] = 161.461 and adjusted *P* value = 7.7 × 10^−8^). For all conditions, butyrate concentrations in coculture after 6 days were 1.1 ± 0.24 mmol · liter^−1^ lower than in monocultures (Fig. 6a to c and Table A3 in Text S1). The interaction factor between the mono/coculture conditions and the growth condition was not significantly correlated with butyrate concentrations (ANOVA, *F* value [2] = 0.862, adjusted *P* value = 0.4). The observation that butyrate concentrations in cocultures were lower than in monocultures regardless of pressure and headspace composition suggest that the methanogen’s presence shapes the metabolite output of *C. minuta*.

We observed, along with the reduced butyrate production, slightly but significantly higher acetate production in cocultures than in monocultures (Fig. 6d to f and 5d) (ANOVA, *F* value [1] = 317.41 and adjusted *P* value = 3.2 × 10^−9^). This difference was also observed in three additional batches performed at 2 × 10^5^ Pa (Fig. S4). The differences in acetate production between mono- and coculture conditions significantly varied with the headspace and pressure conditions (the interaction term between the mono- or coculture and the growth condition was significantly correlated with acetate production; ANOVA, *F* value [2] = 29.09 and adjusted *P* value = 3.0 × 10^−5^). The differences in final acetate production (after 6 days) ranged from +0.7 mmol · liter^−1^ at 2 × 10^5^ Pa under a H_2_-CO_2_ (80:20, vol/vol) atmosphere to +2.2 mmol · liter^−1^ at atmospheric pressure under a N_2_-CO_2_ (80:20, vol/vol) atmosphere. Furthermore, we observed in coculture more CH_4_ than what *M. smithii* could have produced based on the H_2_ production in *C. minuta*’s monocultures (see Appendix 3 in Text S1). This observation implies that *C. minuta* likely produced a greater amount of H_2_ in the cocultures, along with greater acetate production, than in monocultures.

10.1128/mBio.03235-19.4 FIG S4 Additional batches. H_2_, CH_4_, acetate, and butyrate concentrations in mono- and cocultures of *M. smithii* and *C. minuta* grown at 2 × 10^5^ Pa, as described in the main text. The SCFAs of batch S1 were measured by gas chromatography instead of high-performance liquid chromatography. The points represent the averages of results from 2 to 3 biological cultures, and red bars join the minimal and maximal values. Download FIG S4, PDF file, 1.1 MB. Copyright © 2020 Ruaud et al. 2020 Ruaud et al. This content is distributed under the terms of the Creative Commons Attribution 4.0 International license.

### *C. massiliensis and C. timonensis* also support the metabolism of *M. smithii*.

**FIG 7:**
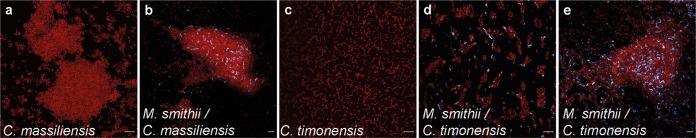
Confocal imaging of *C. massiliensis* and *C. timonensis* in mono- and cocultures with *M. smithii*. Confocal micrographs after 5 days of growth of *C. massiliensis* (a), *M. smithii* and *C. massiliensis* in coculture (b), *C. timonensis* (c), and *M. smithii* and *C. timonensis* in coculture (d and e). SYBR green I fluorescence (DNA staining) is shown in red, and *M. smithii*’s coenzyme F420 autofluorescence is shown in blue. Scale bars represent 10 μm.

**FIG 8:**
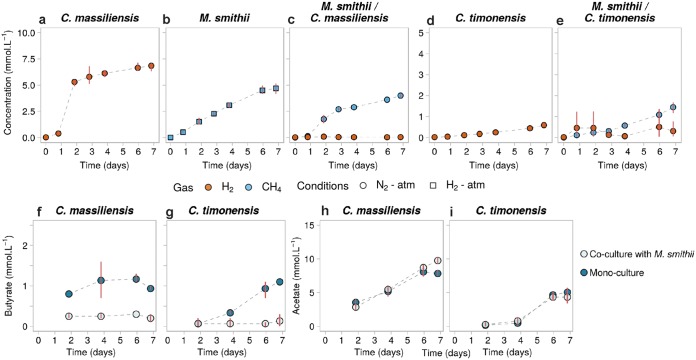
Gas and SCFA concentrations in mono- and cocultures of *C. massiliensis* and *C. timonensis* with *M. smithii*. (a to e) H_2_ (orange) and CH_4_ (blue) concentrations in the headspace of cultures from batch 4 (Table S1); (f to i) butyrate (f and g) and acetate (h and i) concentrations in these cultures. Points represent the averages of results of 3 biological replicates, and red bars join the minimal and maximal values. (b) In the monocultures of *M. smithii* where H_2_ was provided in excess (condition, H_2_ atmosphere, with the headspace initially composed of 80:20, vol/vol, H_2_-CO_2_), its concentrations are not shown for scale reasons.

We performed similar coculture experiments of *M. smithii* with *C. massiliensis* and *C. timonensis* at atmospheric pressure. *C. massiliensis* and *C. timonensis* aggregated in monoculture, and *M. smithii* grew within its flocs in coculture (Fig. 7). The H_2_ produced by the bacteria in monoculture after 6 days of growth (6.9 ± 0.5 mmol · liter^−1^ for *C. massiliensis* and 0.6 ± 0.1 mmol · liter^−1^ for *C. timonensis*) (Fig. 8a and d) was lower than the levels produced by *C. minuta* (Fig. 4a). CH_4_ production in the cocultures reached 4.0 ± 0.2 mmol · liter^−1^ with *C. massiliensis* and 1.5 ± 0.3 mmol · liter^−1^ with *C. timonensis*. These amounts of methane are significantly lower than what we observed for *M. smithii* with *C. minuta* (6.6 ± 0.8 mmol · liter^−1^; ANOVA followed by a Tukey’s *post hoc* test, adjusted *P* values = 6.8 × 10^−2^ and 1.7 × 10^−3^ for cocultures, respectively, with *C. massiliensis* and *C. timonensis* against *C. minuta*) (Fig. 4c and 8c and e).

We observed less butyrate production in the cocultures than in the monocultures (Wilcoxon rank sum test, *P* values = 0.33 for *C. massiliensis* and 0.5 for *C. timonensis*) (Fig. 8f and g), with butyrate measured barely above the detection limit in cocultures. While in monocultures, *C. massiliensis* and *C. timonensis* produced 0.93 ± 0.06 mmol · liter^−1^ and 1.10 ± 0.00 mmol · liter^−1^ of butyrate, respectively; in coculture with *M. smithii*, they produced 0.20 ± 0.14 mmol · liter^−1^ and 0.13 ± 0.15 mmol · liter^−1^, respectively. Acetate production by *C. massiliensis* was higher in coculture than in monoculture (7.83 ± 0.49 mmol · liter^−1^ of acetate produced in monoculture by day 6 and 9.75 ± 0.78 mmol · liter^−1^ produced in coculture with *M. smithii*), although this difference was not significant (Wilcoxon rank sum test, *P* value = 0.2). And in contrast with the cocultures of *C. minuta* with *M. smithii*, acetate production by *C. timonensis* was not higher in the cocultures than in monocultures: *C. timonensis* produced 5.05 ± 0.21 mmol · liter^−1^ in monoculture and 4.33 ± 1.21 mmol · liter^−1^ in coculture (Wilcoxon rank sum test, *P* value = 0.8) (Fig. 8h and i).

## DISCUSSION

The link between the relative abundance of the *Christensenellaceae* and host BMI now stands as one of the most reproducible associations described between the gut microbiome and obesity (4–15). Here, we confirm in a meta-analysis of metagenomes across 10 populations the previously observed association between leanness and the *Christensenellaceae* family (4, 20–22). We could also show that the *Christensenella* genus and *Christensenella* spp. also correlated with leanness. Similarly, we observed correlations between leanness and the *Methanobacteriaceae* family, the *Methanobrevibacter* genus, and *M. smithii*. These methanogens were positively correlated with members of the *Christensenellaceae* family. The relative abundances of the *Christensenellaceae* were higher in young people, whereas conversely, *Methanobacteriaceae* were enriched in older people. Despite these opposite patterns, the two families correlate with each other regardless of age and BMI.

We selected the two most prominent members of the two families, *C. minuta* and *M. smithii*, to ask if physical and metabolic interactions may underlie these positive associations. *C. minuta* produced copious amounts of H_2_ during fermentation. In coculture with *C. minuta*, *M. smithii* produced amounts of CH_4_ comparable to those in monoculture with an excess of H_2_, indicating that *C. minuta* can efficiently support the growth of *M. smithii* via interspecies H_2_ transfer. *C. minuta* formed flocs visible by eye, and *M. smithii* grew within these flocs.

*M. smithii* would likely benefit by associating with the flocs formed by *C. minuta* through better access to H_2_. Interspecies metabolite transfer corresponds to the diffusion of a metabolite (e.g., H_2_) from the producer (e.g., *C. minuta*) to the consumer (e.g., *M. smithii*). As described by Fick’s law of diffusion, the flux of a metabolite between two microorganisms is directly proportional to the concentration gradient and inversely proportional to the distance, such that the closer the microorganisms are, the better the H_2_ transfer (35, 36). Thus, within the flocs, the H_2_ interspecies transfer would be more efficient, to the benefit of *M. smithii*. In accord, we observed greater methane production under excess H_2_ when *C. minuta* was present.

When grown in coculture, *M*. *smithii* influenced the metabolism of *C. minuta*. The presence of the methanogen inhibited the production of butyrate while enhancing acetate production by *C. minuta* under all growth conditions, on average among all experimental batches. This observation suggests that H_2_ consumption by *M. smithii* decreased the P_H2_ within the floc enough to favor acetate production (37). The consumption of H_2_ causes the cell to produce more oxidized fermentation products, such as acetate (38–41), and the interspecies H_2_ transfer leads to greater CH_4_ production.

Both the methane production and the coflocculation were far more pronounced when *M. smithii* was grown with *C. minuta* than with B. thetaiotaomicron. B. thetaiotaomicron has previously been shown to support the growth of *M*. *smithii* in coculture (25, 26). B. thetaiotaomicron barely aggregated, in contrast to *C. minuta*’s very large (visible to the naked eye) flocs. When grown together, B. thetaiotaomicron and *M. smithii* showed very poor aggregation. Moreover, acetate was the only SCFA detected in monocultures of B. thetaiotaomicron, and its production was less affected by the methanogen than in *C. minuta*. Methane produced by *M. smithii* in coculture with B. thetaiotaomicron was one-fifth of that produced with *C. minuta*, possibly as a result of the smaller amount of H_2_ produced and the reduced contact between cells. Given that *M. smithii* does not cooccur with B. thetaiotaomicron in human microbiome data sets, this is another indication that cooccurrence patterns may point to metabolic interactions.

*C. massiliensis* and *C. timonensis* also produced H_2_, acetate, and butyrate and also flocculated in monoculture. *C. massiliensis* and *C. timonensis* supported methane production by *M. smithii*, which grew within the bacterial flocs. However, *M. smithii* also grew outside the flocs when cocultured with these two species, which we did not observe in the cocultures with *C. minuta*, and although *M. smithii* also influenced the fermentation of *C. massiliensis* and *C. timonensis*, the overall changes in SCFA production in coculture were different from what we observed with *C. minuta*: butyrate production was almost undetectable, while acetate production was not significantly affected.

These results suggest that the interaction between *M. smithii* and *C. minuta* leads to higher methane production than with B. thetaiotaomicron and other species of the *Christensenellaceae*, possibly due to the higher levels of interspecies H_2_ transfer. Nevertheless, *C. massiliensis* and *C. timonensis* supported CH_4_ production better than B. thetaiotaomicron did. The higher H_2_ production of *C. massiliensis* than of B. thetaiotaomicron might explain this. In the case of *C. timonensis*, although it produced half the H_2_ produced by B. thetaiotaomicron in monoculture, *M. smithii* produced more CH_4_ in coculture with *C. timonensis* than with B. thetaiotaomicron. This suggests that, as with its effect on *C. minuta*, *M. smithii* triggered the production of H_2_ by *C. timonensis*.

Altogether, our work demonstrates that members of the *Christensenellaceae* act as a H_2_ source to methanogens, and this process is enhanced via close physical proximity. Such interactions also likely underlie the cooccurrence patterns of the *Christensenellaceae* with other members of the microbiome. Many of these families lack cultured representatives, such as *Firmicutes* unclassified SHA-98, *Tenericutes* unclassified RF39, and unclassified ML615J-28 (4). Based on our results, cultivation of these elusive members of the microbiome may require H_2_ (or the provision of another metabolite that *C. minuta* produces when H_2_ is being consumed). Despite their very low abundance in the human gut, members of the *Christensenellaceae* may shape the composition of the gut microbiome by favoring the colonization and persistence of certain hydrogenotrophs and by supplying other butyrate producers with acetate (42).

Here, we confirmed an association of *M. smithii* and leanness based on metagenomes from 10 studies. In contrast, some studies have reported an association between *M. smithii* and obesity (2, 43). In this scenario, H_2_ uptake by *M. smithii* would promote the breakdown of nondigestible carbon sources by fermenters, such as acetogens, thereby increasing the amount of acetate or other SCFAs that can be absorbed and utilized by the host and promoting fat storage (2, 44). In contrast, and consistently with our results, *M. smithii* has also been repeatedly associated with anorexia and leanness (4, 45–48). In this case, the production of CH_4_ would decrease the amount of energy available for the host via carbon loss, as has been observed in livestock (49–52). Thus, our observation that the presence of *M. smithii* directs the metabolic output of the *C. minuta* toward greater H_2_ availability for methanogenesis via increased acetate production is consistent with their association with a lean phenotype. To assess quantitatively how the presence and activity of these microbes impact host physiology will require careful modeling of energy flow *in vivo*.

## MATERIALS AND METHODS

### Metagenome data generation.

We generated 141 metagenomes from fecal samples obtained as part of a previous study (53) (see Table S2 in the supplemental material). Metagenomic libraries were prepared as described in Appendix 1 (additional methods) in Text S1.

10.1128/mBio.03235-19.6 TABLE S2 Samples from the Poole et al. (53) study. Additional data were generated from time points that had not been sequenced for the Poole et al. study. For each individual and each time point, the color indicates whether the sample was prepared and sequenced as described previously (orange) or as described in Materials and Methods (blue). If the sample failed sequencing or was otherwise missing, the color is white. Download Table S2, PDF file, 0.04 MB. Copyright © 2020 Ruaud et al. 2020 Ruaud et al. This content is distributed under the terms of the Creative Commons Attribution 4.0 International license.

### Data from public databases.

We constructed a metagenome sequence collection from (i) the newly generated data (above) to complement the 146 metagenomes previously reported by Poole et al. in 2019 (53) and (ii) publicly available shotgun-metagenome sequences from stool samples included in the curatedMetagenomicData package of Bioconductor (54) for which BMI information was provided. For the latter, we restricted our analyses to individuals for which the following information was available: gender, age, country of origin, and BMI. Individuals with *Schistosoma* (*n* = 4) or Wilson's disease (*n* = 2) were excluded from the analysis, as were samples from two pregnant women. In all, 1,534 samples from 9 studies were downloaded from the sequence read archive (SRA) and further processed (Table S3), for a total or 1,821 samples with at least 1 million sequence pairs per sample.

### Data processing.

A detailed description of the processing of the raw sequences is given in Appendix 1 in Text S1. To obtain a taxonomic profile of the metagenome samples, we built a custom genome database (55) for Kraken v2.0.7 (56) and Bracken v2.2 (57) using the representative genomes from the proGenomes database (as available on 24 August 2018) (58), to which we added genome sequences of *C. minuta* (GenBank assembly accession number GCA_001652705.1), *C. massiliensis* (GCA_900155415.1), and *C. timonensis* (GCA_900087015.1). Reads were classified using Kraken2, and a Bayesian reestimation of the species-level abundance of each sample was then performed using Bracken2. We obtained complete taxonomic annotations from NCBI taxon IDs with TaxonKit v0.2.4 (https://bioinf.shenwei.me/taxonkit/). The detection limit for the relative abundances in samples was 10^−3^%; in consequence, all relative abundances below this threshold were equal to 0.

### Meta-analysis of human gut metagenomes.

Linear mixed models (R package nlme) were used to evaluate the correlation between the relative abundances of taxa while correcting for the structure of the population; the study of origin was set as a random effect. In some data sets, individuals were sampled multiple times, in which case the individual effect was nested inside the data set effect. Relative abundances were transformed using Tukey's ladder of power transformation (59) and are designated with the suffix “-tra” (e.g., the transformed relative abundance of the family *Christensenellaceae* is Cf-tra). Covariates in null models were selected using a backward feature selection approach based on a type II ANOVA (i.e., by including all covariates and removing the nonsignificant ones step-by-step until all remaining variables were significant [see Appendix 2 in Text S1). We made 4 null models predicting the transformed relative abundances of members of the family *Christensenellaceae* (Cf-null), the genus *Christensenella* (Cg-null), the family *Methanobacteriaceae* (Mf-null), and the genus *Methanobrevibacter* (Mg-null). To evaluate the correlation between taxa, we made model Cf-Mf by adding Mf-tra and its interaction with age to the covariates of Cf-null. Reciprocally, we made model Cg-Mg by adding Mg-tra and its interaction with age to the covariates of Cg-null. The same approach was performed at the species level, and it is described in Appendix 2 in Text S1.

For Cf-null, Cf-tra=BMI + age + 1|data set/individual

For Cg-null, Cg-tra = BMI + age + 1|data set/individual

For Mf-null, Mf-tra = BMI + age + 1|data set/individual

For Mg-null, Mg-tra = BMI + age + 1|data set/individual

For Cf-Mf, Cf-tra = BMI + age + Mf-tra + age×Mf-tra + 1|data set/individual

For Cg-Mg, Cg-tra = BMI + age + Mg-tra + age×Mg-tra + 1|data set/individual

We used the likelihood ratio test to compare the nested models via the χ^2^ distribution (i.e., Cf-null versus Cf-Mf and Cg-null versus Cg-Mg). To characterize the correlation of Cf-tra with Mf-tra and Cg-tra with Mg-tra, after correcting for BMI and age, we used a type I ANOVA to evaluate the importance of the variables in the order in which they appear in Cf-Mf and Cg-Mg. The *F* value, degree of freedom, and *P* value are reported for each variable. All analyses were performed using R (60).

### Culturing of methanogens and bacteria.

We obtained *M. smithii* DSM-861, *C. minuta* DSM-22607, *C. massiliensis* DSM 102344, *C. timonensis* DSM 102800, and B. thetaiotaomicron VPI-5482 from the German Collection of Microorganisms and Cell Cultures (DSMZ; Braunschweig, Germany). Each culture was thawed and inoculated into brain heart infusion (BHI) medium (Carl Roth, Karlsruhe, Germany) supplemented with yeast extract (5 g/liter), reduced with l-cysteine-HCl (0.5 g/liter) and Ti-NTA III (0.3 mM), and buffered with sodium bicarbonate (42 mM, pH 7, adjusted with HCl 6 M). Cultures (10 ml) were grown at 37°C without shaking in Balch tubes (total volume of 28 ml) under a headspace of N_2_-CO_2_ (80:20, vol/vol) in the case of the bacteria and H_2_-CO_2_ (80:20, vol/vol, with pressure adjusted to 2  × 10^5^ Pa) for *M. smithii*. When initial cultures reached exponential growth and before floc formation, they were transferred into fresh medium, and these transfers were used as inocula for the experiments described below.

### Coculture conditions.

*M. smithii* was cocultured with *C. minuta*, B. thetaiotaomicron, *C. massiliensis*, or *C*. *timonensis*, and in parallel, each microorganism was grown in monoculture (Table S1). Prior to inoculation, 1-day-old cultures of bacterial species or 4-day-old cultures of *M. smithii* were adjusted to an optical density at 600 nm (OD_600_) of 0.01 with sterile medium. For the cocultures, 0.5 ml of each adjusted culture was inoculated into 9 ml of fresh medium. For the monocultures, 0.5 ml of the adjusted culture and 0.5 ml of sterile medium were combined as an inoculum. For negative controls, sterile medium was transferred as a mock inoculum. Headspaces were exchanged with 80:20 (vol/vol) N_2_-CO_2_ or H_2_-CO_2_ and pressurized at 2 × 10^5^ Pa or atmospheric pressure (Table S1). Each batch of experiments was carried out once with 3 biological replicates per culture condition (Table S1).

### Imaging.

For confocal microscopy, SYBR green I staining was performed as previously described (61) with the modifications described in Appendix 1 in Text S1. Imaging by confocal microscopy (LSM 780 NLO; Zeiss) was used to detect the autofluorescence emission of coenzyme F_420_ of *M. smithii* and the emission of SYBR green I (Appendix 1 in Text S1). Images were acquired with the ZEN Black 2.3 SP1 software and processed with FIJI (62). Micrographs are representative of all replicate cultures within each experimental batch. The preparation of the samples for scanning electron microscopy is described in Appendix 1 in Text S1. Cells were examined with a field emission scanning electron microscope (Regulus 8230; Hitachi High Technologies, Tokyo, Japan) at an accelerating voltage of 10 kV.

### Gas and SCFA measurements.

Headspace concentrations of H_2_, CO_2_, and CH_4_ were measured with a gas chromatograph (GC) (SRI 8610C; SRI Instruments, Torrance, USA) equipped with a packed column at 42°C (0.3-m HaySep-D packed Teflon; Restek, Bellefonte, PA, USA), a thermal conductivity detector (TCD) at 111°C, and a flame ionization detector (FID). The gas production and consumption were estimated from the total pressure in the vials (ECO2 manometer; Keller, Jestetten, Germany) and the gas concentrations in the headspace using the ideal gas equation. The concentrations are given in millimoles of gas in the headspace per liter of culture.

SCFA measurements were performed with liquid samples (0.5 ml) filtered through 0.2-μm-pore-size polyvinylidene fluoride filters (Carl Roth, GmbH, Karlsruhe, Germany). SCFA concentrations were measured with a CBM-20A high-performance liquid chromatography (HPLC) system equipped with an Aminex HPX-87P column (300 by 7.8 mm; Bio-Rad, CA, USA), maintained at 60°C, and a refractive index detector. A sulfuric acid solution (5 mM) was used as the eluent at a flow rate of 0.6 ml/min (**∼**40 × 10^5^-Pa column pressure). Calibration curves for acetate and butyrate were prepared from 1.25 to 50 mM using acetic acid and butyric acid, respectively (Merck KGaA, Darmstadt, Germany). No other fatty acids were detected (see Appendix 1 in Text S1 [63–69]). The SCFA concentrations were estimated with the Shimadzu LabSolutions software.

### Statistical analyses.

We used Wilcoxon rank sum tests to compare levels of gas production between cultures after 6 days of growth. We performed ANOVA tests when more than one culture condition (i.e., headspace composition and pressure) (Table S1) was included in the comparison. The conditions in the ANOVA tests (i.e., headspace composition and pressure in mono- or cocultures) were evaluated to explain the variance of CH_4_ production after 6 days of growth. A Tukey *post hoc* test was then performed to discriminate between the effects of the different conditions. SCFA concentrations were compared using a two-way ANOVA where the culture conditions (i.e., headspace composition and pressure) (Table S1) and the sample (mono- and cocultures) were evaluated to explain the variance of butyrate and acetate concentrations after 6 days of growth. *P* values were adjusted using the Benjamini-Hochberg method. Tukey’s *post hoc* test was performed to discriminate between the effects of the different conditions. All statistical analyses were done in R using the stats R package.

### Data availability.

The metagenomic sequence data generated during this study have been deposited in the European Nucleotide Archive under accession number PRJEB34191 (http://www.ebi.ac.uk/ena/data/view/PRJEB34191). The jupyter notebooks and associated data are available at https://github.com/Albabune/Ruaud_EsquivelElizondo.
