# Supplementary figures and images for: Syntrophy via Interspecies H2 Transfer between Christensenella and Methanobrevibacter Underlies Their Global Cooccurrence in the Human Gut
Source: mBio. 2020 Feb 4;11(1):e03235-19. doi: 10.1128/mBio.03235-19 (PMC7002349; doi:10.1128/mBio.03235-19)

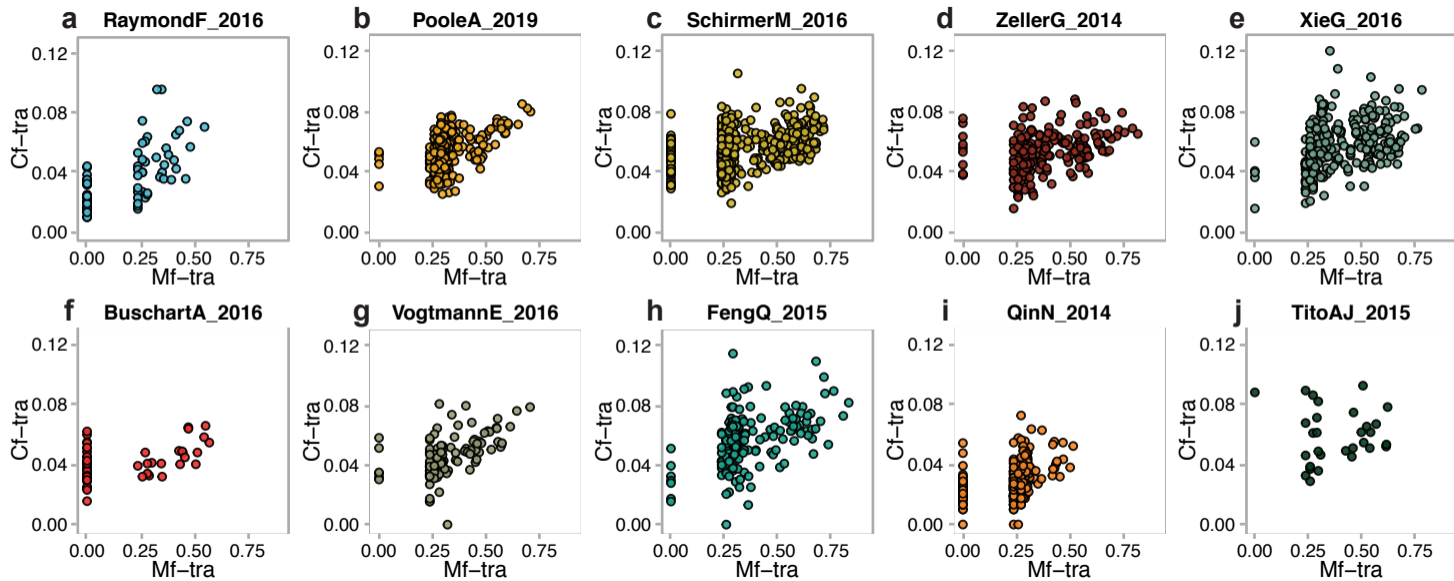

Supplement: FIG S1 [file mBio.03235-19-sf001.pdf]

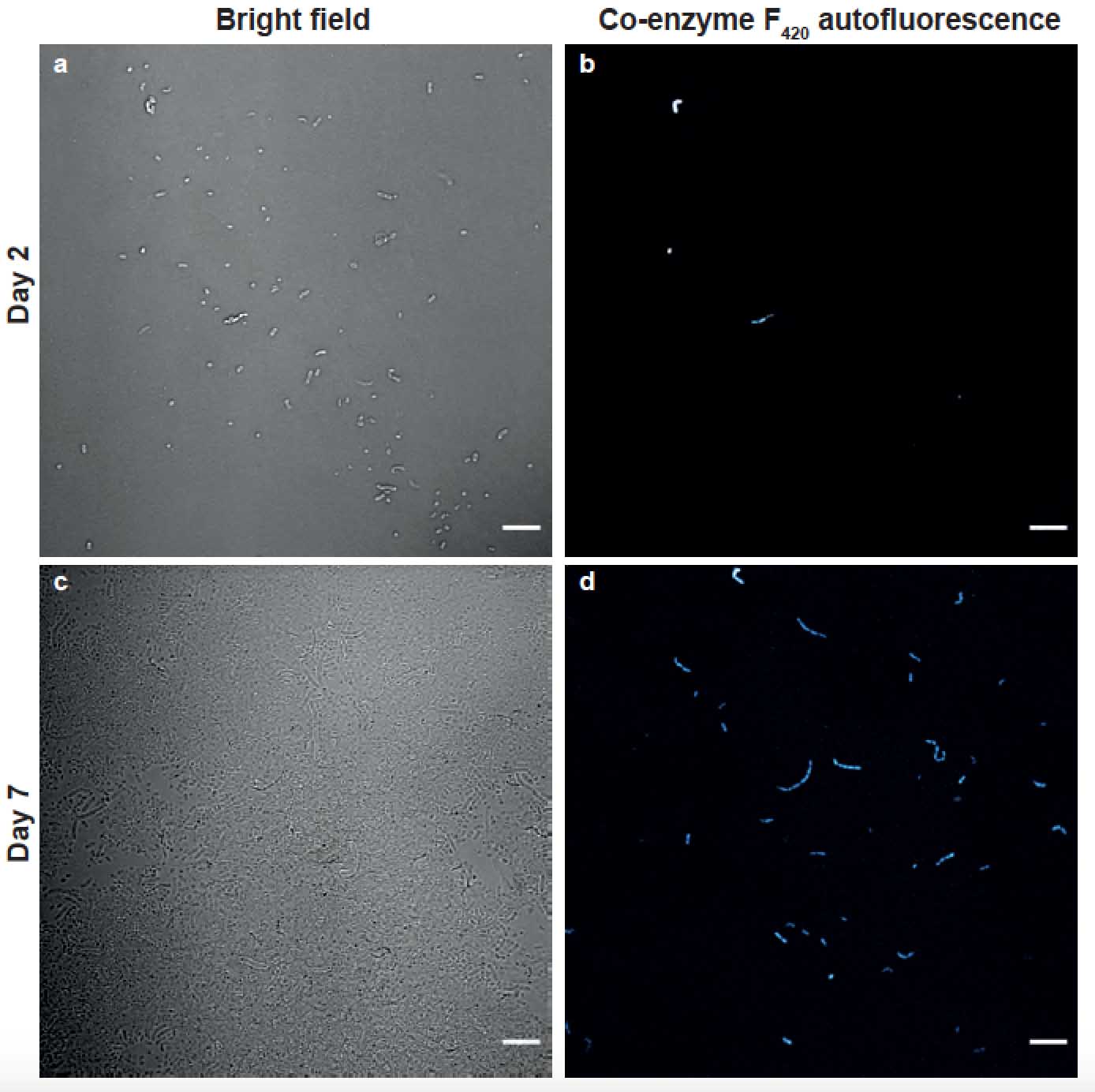

Supplement: FIG S2 [file mBio.03235-19-sf002.jpg]

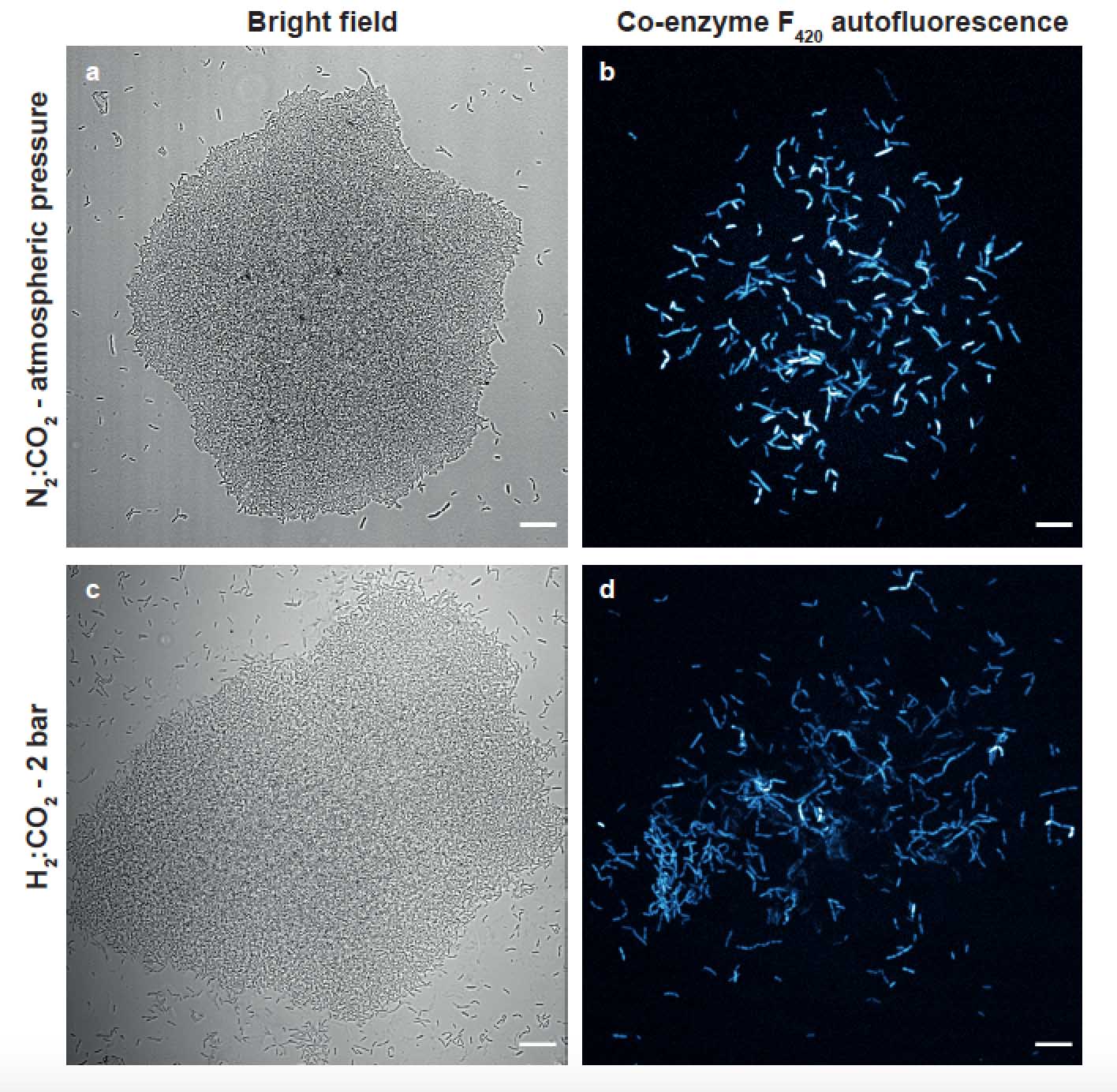

Supplement: FIG S3 [file mBio.03235-19-sf003.jpg]

Batch S1: N<sub>2</sub> - 2 barBatch S2: N<sub>2</sub> - 2 barBatch S3: N<sub>2</sub> - atmBatch S4: N<sub>2</sub> - atm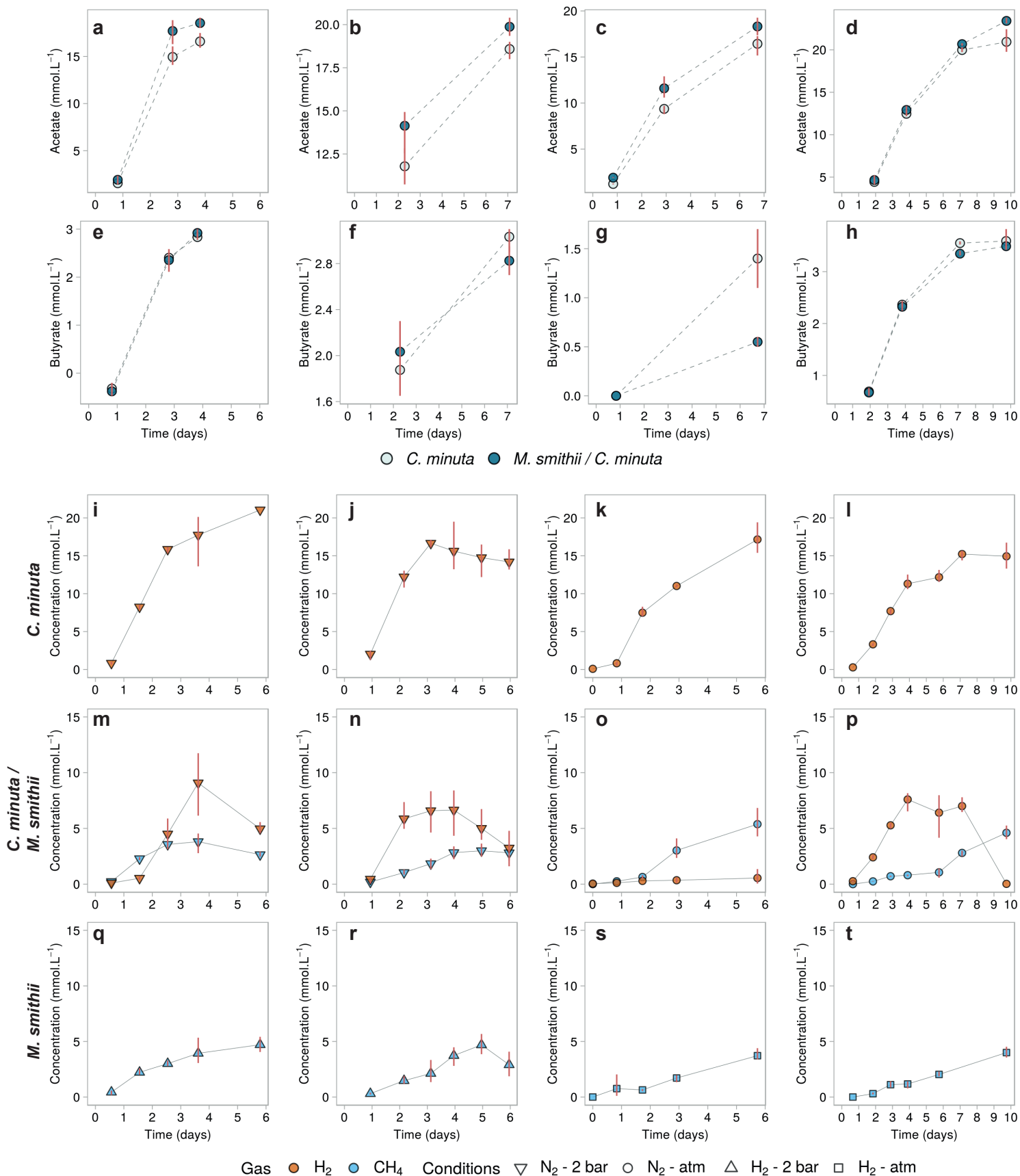

Supplement: FIG S4 [file mBio.03235-19-sf004.pdf]
