# Supplementary material for: Syntrophy via Interspecies H2 Transfer between Christensenella and Methanobrevibacter Underlies Their Global Cooccurrence in the Human Gut
Source: mBio. 2020 Feb 4;11(1):e03235-19. doi: 10.1128/mBio.03235-19 (PMC7002349; doi:10.1128/mBio.03235-19)
Supplement: TEXT S1 [file mBio.03235-19-s0001.docx]

# **Appendixes**

**Appendix 1. Additional methods**

**Metagenomic libraries preparation -** Metagenomic libraries were prepared using 1 ng of DNA input per sample (extracted with the MagAttract PowerSoil DNA kit, Qiagen) as previously described [(63)](https://paperpile.com/c/CqygEn/BTmds). Fragment sizes were restricted to 400 - 700 bp using BluePippin (Sage Science), and samples were pooled at equimolar concentrations before being run on an Illumina HiSeq3000 with 2x150 bp paired end sequencing, resulting in sequencing depths of 3.0 ± 2.1 Gb (median ± standard deviation).

**Raw Data processing -** Raw sequences were first validated using fqtools v.2.0 [(64)](https://paperpile.com/c/CqygEn/ZI8j7) and de-duplicated with the “clumpify” command of bbtools v37.78 (<https://jgi.doe.gov/data-and-tools/bbtools/>). We trimmed adapters and performed read quality control using skewer v0.2.2 [(65)](https://paperpile.com/c/CqygEn/GhS9D) and the “bbduk” command of bbtools. We used the “bbmap” command of bbtools to filter human genome reads that mapped to the hg19 assembly. Finally, we generated QC reports for all reads with fastqc v0.11.7 (https://github.com/s-andrews/FastQC) and multiQC v1.5a [(66)](https://paperpile.com/c/CqygEn/VnzPO).

**Confocal imaging, equipment, and settings -**  For confocal microscopy, SYBR^®️^ Green I staining was performed as previously described [(61)](https://paperpile.com/c/CqygEn/OX6tY) with the following modifications: 0.5 mL of culture were sampled and pelleted by centrifugation for 6 min at 6,000 xg (Benchtop centrifuge, Eppendorf, Hamburg, Germany) and pellets were resuspended in a solution containing 744 μL 1x PBS, 16 μL 25x SYBR^®️^ Green I (Sigma-Aldrich, Merck, Germany) and 40 μL 70% v/v ethanol. Samples were pelleted and resuspended before imagining in 100 μL 1x PBS, of which 5 μL were immobilized on 50 μL solid agar (1.5% noble agar in distilled water) [(67)](https://paperpile.com/c/CqygEn/Z4260). Imaging was performed with a confocal microscope (LSM 780 NLO, Zeiss) using oil and water objectives (40x and 63x). A DPSS laser at 405 nm was used to excite the F_420_ enzyme of *M. smithii.* Autofluorescence emission was collected on a 32 channel GaAsP array from 455 to 499 nm. A transmitted light detector (T-PMT) was used to collect the whole light spectrum to create a bright field image. On a second track, an Argon laser at 488 nm was used to excite SYBR^®️^ Green I and its emission was collected from 508 to 588 nm with the 32 channel GaAsP array as well. Images were acquired with a time and space resolution of 2048x2048x(1 to 12)x (xyzt) and pixel dimensions of 0.1038x0.1038 μm for the images taken with the x40 oil objective and pixel dimensions of 0.0659x0.0659 μm for the images taken with the x63 oil objective. The bit depth was 16-bit. Acquisition was performed at 20 ˚C.

**Processing of the confocal images** - FIJI [(62)](https://paperpile.com/c/CqygEn/XOnzs) was used to process the confocal micrographs. Contrast and brightness adjustment were applied to the whole image. Due to the thickness of the aggregates of *Christensenella minuta*, the SYBR^®️^ Green I fluorescence intensity was varied with different focal planes. We used a gamma transformation (with gamma = 0.50) to homogenize the fluorescence intensity. The exact same transformation was applied to all samples, even though there were no aggregates, for consistency purposes. Similarly, we applied a gamma transformation to the F_420_ autofluorescent channel to decrease the low fluorescence coming from SYBR^®️^ Green I (gamma = 1.20 to 1.50). As their excitation and emission spectra overlap, there was a low fluorescence intensity of the SYBR^®️^ Green I on the F_420_ autofluorescent channel. The lookup tables (LUT) were Cyan Hot for the F_420_ autofluorescence and red (linear LUT, covering the full range of the data) for the SYBR^®️^ Green I fluorescence.

**Preparation of samples for scanning electron microscopy -** Pellets were washed 3-5 times with 1x PBS and then fixed with a 2.5% v/v glutaraldehyde solution in 1x PBS for 1-2 h at room temperature and post-fixed with 1% w/v osmium tetroxide for 1h on ice. Samples were dehydrated in a graded ethanol series followed by drying with CO_2_ in a Polaron critical point dryer (Quorum Technologies, East Sussex, UK). Finally, cells were sputter coated with a 5 nm thick layer of platinum (CCU-010 Compact coating unit, Safematic GmbH, Bad Ragaz, SWI).

**Screening of the short and medium chain fatty acids produced -** Before carrying out the experiments presented in the main text, we used gas chromatography (GC) to determine which fatty acids were produced by the cultures and if the corresponding peaks were present in BHI. For this screening, the external standards included equimolar mixtures of acetate, propionate, iso-butyrate, butyrate, iso-valerate, valerate, iso-caproate, caproate, heptanoate, and caprylate, from 0.2 to 7 mM. Measurements were performed with a 7890B GC system (Agilent Technologies Inc., Santa Clara, USA) equipped with a capillary column (DB-Fatwax UI 30 m x 0.25 m; Agilent Technologies) and an FID detector with a ramp temperature program (initial temperature of 80 ˚C for 0.5 min, then 20 ˚C per min up to 180˚, and final temperature of 180 ˚C for 1 min). The injection and detector temperatures were 250 and 275 ˚C, respectively. Samples were prepared as for HPLC (Methods in the main text) with the addition of an internal standard (Ethyl-butyric acid) and acidification (to pH 2) with 50% formic acid. Data were acquired and analysed with the Agilent OpenLAB CDS software.

Only acetate and butyrate were detected in the mono- and co-cultures, and none of the other short and medium chain fatty acids used as standards were detected. As formate was used to acidify samples for the GC measurements, to assess if it was a main product in the cultures, its concentration was measured by HPLC. We also looked for ethanol using HPLC but similar to formate, it was not detected in any of the cultures.Thus, for the experiments in the main text, only acetate and butyrate were quantified via HPLC. BHI medium showed peaks corresponding to 0.33 mM formate and 6 mM of acetate, which were subtracted from the reported concentrations of the cultures.

**Appendix 2. Additional statistics**

**Variable selection for the null model -** To construct the null model, we tested the effect of the following covariates with a marginal ANOVA: sequencing depth, gender, country, BMI, and age. The sequencing depth was not significant (p-value = 0.73) and was subsequently removed. BMI and age were correlated with the *Chistensenellaceae* abundance (p-value = 0.0002 for the correlation with BMI and 0.02 for the correlation with age).

As *Methanobrevibacter smithii* has been associated with age [(23)](https://paperpile.com/c/CqygEn/hVRtY) and BMI [(4, 43, 45–48, 68, 69)](https://paperpile.com/c/CqygEn/wFD5z+DRUBO+FdmXz+Yaid+zicAa+VgfNI+RAyh2+8afrt), we first added the interaction factors to the models: the interaction factors were not significant with BMI (p-values = 0.11 and 0.07, for both *Methanobrevibacter* and *Methanobacteriaceae*, respectively). However, the interactions with age were significant (p-values = 0.004 and 0.002, for both *Methanobrevibacter* and Methanobacteriaceae, respectively) and therefore, both variables were kept in the models.

**Statistical analysis at the species rank -** Similar to the analysis at family and genus levels presented in the main text, we performed an analysis at the species level between *Christensenella minuta* and *Methanobrevibacter smithii*, the most abundant and prevalent species of their genera. We also studied the correlation of the other two known species of *Christensenella*, *i.e*., *C. massiliensis* and *C. timonenesis*, with *M. smithii*. *M. smithii* was detected in 78.7 % of the samples with a mean relative abundance of 0.53 % (*M. oralis* was the only other *Methanobrevibacter* detected, with a prevalence of 42.8 % and a mean relative abundance of 3.07x10^-3^ %). *C. minuta* had an averaged relative abundance of 0.05% in the 99.7% samples where it was present. *C. timonensis* and *C. massiliensis* had respectively, prevalences of 95.11 % and 98.57 % and mean relative abundances of 6.49x10^-3^ % and 0.02 %.

*M. smithii* was significantly positively correlated with age (type II ANOVA, F-value = 13.22 and p-value = 2.86x10^-4^) and negatively correlated with BMI (type II ANOVA, F-value = 4.13 and p-value = 0.04). The association between *M. smithii* and leanness was not as strong as for its family and genus levels, meaning that other *Methanobacteriaceae* members must contribute to the strength of the association.

Consistently with the analyses at the family and genus levels, *Christensenella minuta* and *Methanobrevibacter smithii* were significantly correlated (χ^2^ test, p-value = 1.05x10^-34^) and the effect of *M. smithii* was significant (type I ANOVA, p-value < 0.0001, F-value = 147.82). Moreover, *C*. *minuta*’s relative abundance correlated with both age and BMI (type I ANOVA, p-values = 0.0071 and 0.0011, F-values = 7.28 and 10.91, respectively), as well as with the interaction term between *M. smithii* and age (type I ANOVA, p-value < 0.0001, F-value = 17.99).

*Christensenella timonensis* and *M. smithii* were correlated (χ^2^ test, p-value = 6.12x10^-98^; type I ANOVA, p-value < 0.0001, F-value = 482.42). And, similar to *C. minuta*, the relative abundance of *C. timonensis* correlated with both age and BMI (type I ANOVA, p-values = 0.0012 and 0.0001, F-values = 10.59 and 16.61, respectively), as well as with the interaction term between *M. smithii* and age (type I ANOVA, p-value < 0.0001, F-value = 35.50).

The relative abundance of *Christensenella massiliensis* correlated with BMI (type I ANOVA, p-value = 0.0028 and F-value = 9.0804) but not with age (p-value > 0.5) and so, we did not correct for age in the null model nor in the model including *Methanobrevibacter smithii*’s transformed relative abundance. *C. massiliensis* and *M. smithii* were also correlated (χ^2^ test, p-value = 1.31x10^-61^; type I ANOVA, p-value < 0.0001, F-value = 310.51), but the interaction term between *M. smithii* and age was not significantly correlated to the bacterium’s abundance. *C. massiliensis* is thus the only *Christensenella* spp. for which the correlation with the methanogen abundance is not a function of the age of the carrier.

**Appendix 3. Comparison of the expected (theoretical) *vs*. the measured methane production in co-cultures**

We used the stoichiometry of hydrogenotrophic methanogenesis (CO_2_ + 4 H_2_ = CH_4_ + 2 H_2_O) to calculate the amount of CH_4_ that could be produced from the estimated amount of H_2_ consumed in each sample. For this, we used the mono-cultures of bacteria as references and assumed equal H_2_ production in co-culture as in mono-culture. We estimated the H_2_ consumed after 6 days for each replicate as the difference between the averaged H_2_ concentrations in mono-cultures and the concentration measured in co-culture (*i*.*e*., unconsumed H_2_). The estimated H_2_ consumed was then divided by 4 in order to obtain the theoretical amount of CH_4_ that could be produced via hydrogenotrophic methanogenesis.

**Table A3. Analysis of the origin of the high methane produced in co-culture based on the changes in metabolism of *C. minuta*.** CH_4_ produced in co-culture was higher than the theoretical amount of CH_4_ that could be generated from H_2_ assuming that *C. minuta* produced the same amount of H_2_ in both mono- and co-cultures. The additional CH_4_ observed could originate from the shift in metabolism from butyrate to acetate production along with H_2_ by *C. minuta* in co-culture. The average concentration among the triplicates after 6 days of growth is given with the standard deviation (SD).

| Condition | H_2_:CO_2_ - 2 bar ^a^ | | N_2_:CO_2_ - atm | | N_2_:CO_2_ - 2 bar | |
| --- | --- | --- | --- | --- | --- | --- |
|  | Average | SD | Average | SD | Average | SD |
| H_2_ produced in mono-culture (mmol.L^−1^) | 18.71 | 9.71 | 17.28 | 1.12 | 14.15 | 1.56 |
| H_2_ not consumed in co-culture ^b^ (mmol.L^−1^) | -21.81 | 0.87 | 0.08 | 0.01 | 0.03 | 0.00 |
| Theoretical CH_4_ produced based on H_2_ produced in mono-culture (mmol.L^−1 c^) | 10.13 | 0.22 | 4.30 | 0.00 | 3.53 | 0.00 |
| Observed CH_4_ produced in co-culture (mmol.L^−1^) | 14.21 | 5.33 | 6.57 | 0.77 | 5.81 | 0.45 |
| Difference between observed and theoretical CH_4_ (mmol.L^−1^) | 04.08 | 5.50 | 2.27 | 0.77 | 2.28 | 0.45 |
| Butyrate difference between co- and mono-culture (mmol.L^−1^) | -1.11 | 0.30 | -1.21 | 0.04 | -0.91 | 0.27 |
| Acetate difference between co- and mono-culture (mmol.L^−1^) | 0.68 | 0.10 | 2.20 | 0.22 | 1.36 | 0.17 |

^a^ For the experiments grown under an H_2_:CO_2_ (80:20 %) atmosphere, the average H_2_ concentration measured in the negative controls after 6 days (sampled as many times as the cultures) was subtracted from the concentration measured in the cultures.

^b^ Average of the concentration of H_2_ in co-cultures to which the average of H_2_ concentration in mono-culture of *C. minuta* was subtracted.

^c^ This amount is calculated based on the stoichiometry of the hydrogenotrophic methanogenesis reaction: 4 H_2_ + CO_2_ = CH_4_ + 2 H_2_O.
