# Supplementary material for: Syntrophy via Interspecies H2 Transfer between Christensenella and Methanobrevibacter Underlies Their Global Cooccurrence in the Human Gut
Source: mBio. 2020 Feb 4;11(1):e03235-19. doi: 10.1128/mBio.03235-19 (PMC7002349; doi:10.1128/mBio.03235-19)
Supplement: TABLE S3 [file mBio.03235-19-st003.pdf]

Designation and Reference	Number of samples	Country	BMI	Age	%♀
Raymond_2016<br>Raymond F, Ouameur AA, Déraspe M, Iqbal N, Gingras H, Dridi B, Leprohon P, Plante P-L, Giroux R, Bérubé È, Frenette J, Boudreau DK, Simard J-L, Chabot I, Domingo M-C, Trottier S, Boissinot M, Huletsky A, Roy PH, Ouellette M, Bergeron MG, Corbeil J. 2016. ISME J 10:707–720	72	CAN	23.97<br>(19.03 - 29.76)	25<br>(21 - 35)	54.17
Buschart_2016<br>Heintz-Buschart A, May P, Laczny CC, Lebrun LA, Bellora C, Krishna A, Wampach L, Schneider JG, Hogan A, de Beaufort C, Wilmes P. 2016. Nat Microbiol 2:16180.	51	LUX	24.68<br>(14.01 - 37.21)	33<br>(5 - 62)	62.75
Poole_2019<br>Poole AC, Goodrich JK, Youngblut ND, Luque GG, Ruaud A, Sutter JL, Waters JL, Shi Q, El-Hadidi M, Johnson LM, Bar HY, Huson DH, Booth JG, Ley RE. 2019. Cell Host Microbe 25:553–564.e7.	287	USA	23.04<br>(19.21 - 28.33)	24<br>(19 - 34)	66.9
Vogtmann_2016<br>Vogtmann E, Hua X, Zeller G, Sunagawa S, Voigt AY, Hercog R, Goedert JJ, Shi J, Bork P, Sinha R. 2016. PLoS One 11:e0155362.	101	USA	25.12<br>(16.83 - 38.37)	61<br>(31 - 89)	27.72
Schirmer_2016<br>Schirmer M, Smeekens SP, Vlamakis H, Jaeger M, Oosting M, Franzosa EA, Ter Horst R, Jansen T, Jacobs L, Bonder MJ, Kurilshikov A, Fu J, Joosten LAB, Zhernakova A, Huttenhower C, Wijmenga C, Netea MG, Xavier RJ. 2016. Cell 167:1125–1136.e8.	456	NLD	22.7<br>(15.09 - 34.42)	28<br>(18 - 75)	57.02
Feng_2015<br>Feng Q, Liang S, Jia H, Stadlmayr A, Tang L, Lan Z, Zhang D, Xia H, Xu X, Jie Z, Su L, Li X, Li X, Li J, Xiao L, Huber-Schönauer U, Niederseer D, Xu X, Al-Aama JY, Yang H, Wang J, Kristiansen K, Arumugam M, Tilg H, Datz C, Wang J. 2015. Nat Commun 6:6528.	154	AUT	27.39<br>(17.99 - 43.58)	67<br>(43 - 86)	43.51
Zeller_2014<br>Zeller G, Tap J, Voigt AY, Sunagawa S, Kultima JR, Costea PI, Amiot A, Böhm J, Brunetti F, Habermann N, Hercog R, Koch M, Luciani A, Mende DR, Schneider MA, Schrotz-King P, Tournigand C, Tran Van Nhieu J, Yamada T, Zimmermann J, Benes V, Kloor M, Ulrich CM, von Knebel Doeberitz M, Sobhani I, Bork P. 2014. Mol Syst Biol 10:766.	194	FRA;<br>DEU	25.58<br>(15 - 40)	62<br>(25 - 90)	40.72

Qin_2014<br>Qin N, Yang F, Li A, Prifti E, Chen Y, Shao L, Guo J, Le Chatelier E, Yao J, Wu L, Zhou J, Ni S, Liu L, Pons N, Batto JM, Kennedy SP, Leonard P, Yuan C, Ding W, Chen Y, Hu X, Zheng B, Qian G, Xu W, Ehrlich SD, Zheng S, Li L. 2014. Nature 513:59–64.	226	CHN	22.25<br>(15.94 - 35.16)	46<br>(18 - 78)	33.63
Xie_2016<br>Xie H, Guo R, Zhong H, Feng Q, Lan Z, Qin B, Ward KJ, Jackson MA, Xia Y, Chen X, Chen B, Xia H, Xu C, Li F, Xu X, Al-Aama JY, Yang H, Wang J, Kristiansen K, Wang J, Steves CJ, Bell JT, Li J, Spector TD, Jia H. 2016. Cell Syst 3:572–584.e3.	250	UK	25.75<br>(16.17 - 44.83)	61<br>(35 - 79)	100
Tito_2015<br>Obregon-Tito AJ, Tito RY, Metcalf J, Sankaranarayanan K, Clemente JC, Ursell LK, Zech Xu Z, Van Treuren W, Knight R, Gaffney PM, Spicer P, Lawson P, Marin-Reyes L, Trujillo-Villarroel O, Foster M, Guija-Poma E, Troncoso-Corzo L, Warinner C, Ozga AT, Lewis CM. 2015. Nat Commun 6:6505.	30	USA; PER	23.42<br>(14.05 - 31.53)	28<br>(3 - 50)	50
