# Supplementary material for: Syntrophy via Interspecies H2 Transfer between Christensenella and Methanobrevibacter Underlies Their Global Cooccurrence in the Human Gut
Source: mBio. 2020 Feb 4;11(1):e03235-19. doi: 10.1128/mBio.03235-19 (PMC7002349; doi:10.1128/mBio.03235-19)
Supplement: TABLE S1 [file mBio.03235-19-st001.pdf]

Batch	Pressure, bar	Headspace composition, 80:20 % v/v	Culture
1	2.0	N_2:CO_2	*C. minuta*
			M. smithii / C. minuta
			B. thetaiotaomicron
			M. smithii / B. thetaiotaomicron
			negative control
		H_2:CO_2	*M. smithii*
			negative control
2	0.98	N_2:CO_2	*C. minuta*
			M. smithii / C. minuta
			negative control
		H_2:CO_2	*M. smithii*
			negative control
3	2.0	H_2:CO_2	*C. minuta*
			M. smithii / C. minuta
			M. smithii
			negative control
4	0.98	N_2:CO_2	*C. massiliensis*
			M. smithii / C. massiliensis
			C. timonensis
			C. timonensis / M. smithii
			negative control
		H_2:CO_2	*M. smithii*
			negative control
